# The fault in our SAAR: optimization and implementation of health-system dashboards for antimicrobial use and SAAR data

**DOI:** 10.1017/ash.2025.10050

**Published:** 2025-11-05

**Authors:** Hunter Odell Rondeau, Kristi Killelea, Meredith B. Oliver, Kimberly Boeser

**Affiliations:** 1 Department of Pharmacy, M Health Fairview University of Minnesota Medical Center, Minneapolis, MN, USA; 2 Department of Pharmacy, M Health Fairview Masonic Children’s Hospital, Minneapolis, MN, USA; 3 Information Technology, M Health Fairview, Minneapolis, MN, USA

## Abstract

**Objective:**

This paper describes a health-system’s experience incorporating Standardized Antimicrobial Administration Ratio (SAAR) data to optimize the existing antimicrobial use module within the electronic health record. We describe the design and implementation via a standard operating procedure (SOP), which incorporates SAAR and the optimized dashboards into the health-system’s antimicrobial stewardship program.

**Design:**

This is a descriptive study outlining our process of optimizing the electronic health records default antimicrobial use dashboard into a dual-purpose dashboard, SAAR and Antimicrobial Use (AU) dashboard, to be used across a health-system.

**Setting:**

10-hospital health-system

**Intervention:**

For adult and pediatric populations, a pre-populated dashboard was created for each SAAR antimicrobial category using the existing EHR application. For adult, pediatric, and neonatal populations, improvements were made to visualize AU data in the dashboards. We created the framework for a standardized metric for our health-system’s antimicrobial stewardship program (ASP). An SOP was developed to establish an expectation for SAAR data analysis at the site level of our health-system’s ASP. The dashboards and SOP were created to evolve with the AUR protocol and health-system.

**Results:**

Pre-populated dashboards for adult and pediatric SAAR antimicrobial categories are configurable within existing electronic health-records, requiring minimal manipulation from the user to view which antimicrobials are driving a location’s SAAR.

**Conclusion:**

In anticipation of required AUR reporting, other health-systems using the same EHR can utilize this approach to integrate the SAAR into their health-system’s antimicrobial stewardship programs. Collaboration between antimicrobial stewardship clinicians, pharmacist information technology (IT) analysts and infection preventionists are essential to accomplish this endeavor.

## Introduction and literature review

### Background and significance of standardized antimicrobial administration ratios (SAAR)

Identifying an appropriate method for collecting and comparing antimicrobial use between hospitals is an ongoing development.^1^ The Standardized Antimicrobial Administration Ratio or SAAR was first provided to hospitals voluntarily participating in the National Health Safety Network Antimicrobial Use (NHSN AU) option in 2015.^2^ The SAAR is an observed-to-predicted ratio and can only be obtained for patient care units that are SAAR-eligible.^3^ Endorsed by the National Quality Forum, the SAAR’s purpose is to provide a quantitative foundation for the NHSN’s antimicrobial use clinical quality measures. Following submission, AU module data are reported back as a SAAR report. The goal of SAAR is to provide a risk-adjusted inter- and intra-facility antimicrobial use benchmarking and monitoring antimicrobial use trends over time at the local, state and national levels. Both the revised 2023 Joint Commission requirements for hospital-based antimicrobial stewardship programs and requirement for all acute-care and critical-access hospitals to report to the NHSN Antimicrobial Usage and Resistance (AUR) module in 2024 will cause a significant increase in the number of hospitals with access to SAAR reports. As SAAR reports become more common, so will the challenge for SAAR to produce actionable value within antimicrobial stewardship programs (ASP’s).^8,9^

### Current challenges in visualizing SAAR data in healthcare

Data visualization tools improve SAAR ratio integration into an ASP.^5^ As outlined by Butterfield- Cowper’s article, a days of therapy (DOT) report in combination with the associated SAAR is better than SAAR alone when prioritizing antimicrobial stewardship efforts and tracking ASP interventions.^14^ Due to the current reporting process, antimicrobial stewardship practitioners find it challenging to easily review, analyze and clinically apply their own SAAR data. A survey of members of the Society of Infectious Diseases Pharmacists and Society of Healthcare Epidemiology of America concluded a significant area of need and key next step for SAAR is to improve the utility and accessibility of AUR analytic tools.^7^ Some electronic health records (EHR) can submit AU data directly to NHSN,^10^ while others submit the data individually. These data are submitted in a file type known as a Clinical Document Architecture or CDA, which are not easily accessible nor transformable.

Some ASP’s have resorted to creating “homegrown” visualization strategies using third party platforms.^5^ While some electronic health records and software providers have tools to visualize antimicrobial use, it is extensively time consuming to interrogate the individual antimicrobials driving abnormal SAAR’s across the many patient care units and hospitals within a health-system.^5–7^ This leads to inefficiency in antimicrobial stewardship action and inability to benchmark data across an organization. In addition, SAAR highlights the areas of variance and deviation from the expected, but it must be combined with clinical context to develop effective antimicrobial stewardship interventions. Our vendor developed a module that allows visualization of both SAAR and DOTs for each reporting location. However, the base configuration of this EHR’s module is not designed exclusively for SAAR interpretation. Therefore, extensive configuration and manipulation is required to summarize data and glean actionable data from the module.

### Purpose and objectives of the manuscript

This paper describes the development and implementation of dashboards within the electronic health record in a 10-hospital health-system by configuring the EHR’s existing module to pre populated SAAR antimicrobial category dashboards. The goals of the dashboards are to provide healthcare professionals with user-friendly, comprehensive, and real-time data on SAAR metrics, enabling the end user to make informed decisions about antimicrobial use by displaying the SAAR and corresponding individual antimicrobial days of therapy (DOT) of a hospital, unit or similar units into one condensed dashboard per antimicrobial category.

## Implementation

### Project scope

Our health-system, comprising two academic (adult and children) and eight community hospitals representing over 2,000 beds, has been submitting AU data to NHSN since 2017, when the antimicrobial stewardship director and IT analyst performed the initial validation. From project conception to release of the customized dashboards took eight months, led by a pharmacy resident with direct mentorship by the system ASP pharmacy manager and pharmacy IT analyst. Specifically, our analyst required 40 hours with an estimated project cost of $3,800 USD. We began by identifying a member of the antimicrobial stewardship team to lead the dashboard development and coordinate the build, testing and education. Collaboration between a system pharmacist IT analyst, system infection preventionist and site antimicrobial stewardship practitioners (infectious diseases physician or pharmacist) was essential to creating these dashboards. From the perspective of our IT analyst, our system’s EHR provider, Epic Systems, had foundational and basic functionality for creating a SAAR dashboard with the Bugsy module, an application focused on antimicrobial stewardship and infection control, that end-user clinicians could use. SAAR calculations are built out in Bugsy as standard functionality following configuration by a pharmacy IT analyst. The project was scoped for all hospitals in the health-system but was piloted at the flagship academic medical center. A customized copy of the foundational dashboard for each SAAR antimicrobial category was created. While the foundational dashboard allowed for hospital and unit level analysis of SAAR data, it required extensive manipulation to drill down to more specific data points. More information about the foundational dashboard’s functionality is available to Epic users on Epic’s UserWeb. To further aid in drilling down into the SAAR data, additional elements were configured to delineate the individual antimicrobial agents that are evaluated in a particular SAAR category, and the individual utilization by agent and unit. By default, the data evaluates back 12 months, however filters can be applied to edit the data ranges. With the creation of these dashboards, we partnered with infection prevention to evaluate all NHSN reportable units to ensure appropriate unit mapping.

### Dashboard creation

Our EHR vendor’s foundation configuration was modified to create custom dashboards based on the Bugsy© Antimicrobial Usage Trending Dashboard. Dashboard development and optimization was centered around two customizations. We first created consolidations of data or “groupers” for the NHSN-defined antimicrobial categories (eg, broad spectrum antibacterial agents predominantly used for hospital-onset infections or BSHO, broad spectrum antibacterial agents predominantly used for community-acquired infections or BSCA, and narrow spectrum beta-lactam agents or NSBL) and locations that are eligible to calculate SAARs (eg, medical critical care, surgical critical care and adult medical wards). Antimicrobial category and reporting location groupers were created for all adult, pediatric and neonatal categories, and SAAR- eligible reporting locations. Second, the dashboard for each SAAR antimicrobial category was built to prepopulate with the corresponding antimicrobial category groupers and associated antimicrobial DOT data. This allows the user to select individual or multiple reporting locations of interest and visualize the individual antimicrobials driving the location’s SAAR. At baseline, the EHR software can calculate a SAAR when parameters are selected. Our health-system took this existing structure and built custom features to enhance visualization of SAAR data This allows end users to create site-specific versions of each dashboard that can be shared amongst ASP team members.

### Resource document creation

A resource document, Appendix 1, was created to assist ASP site leads in utilizing the dashboards and initiating discussion with infection preventionists and pharmacy IT analysts. This document orients the user to AUR protocol definitions before providing an example of how to utilize and interpret the new dashboards. The resource document includes a review of the limitations and key considerations around the utilization of the dashboards. Following a live demonstration of dashboard navigation, the resource document and dashboards were made available to all antimicrobial stewardship clinicians.

## Results

### Overview of the optimized dashboard for visualizing SAAR data

**Figure 1.**
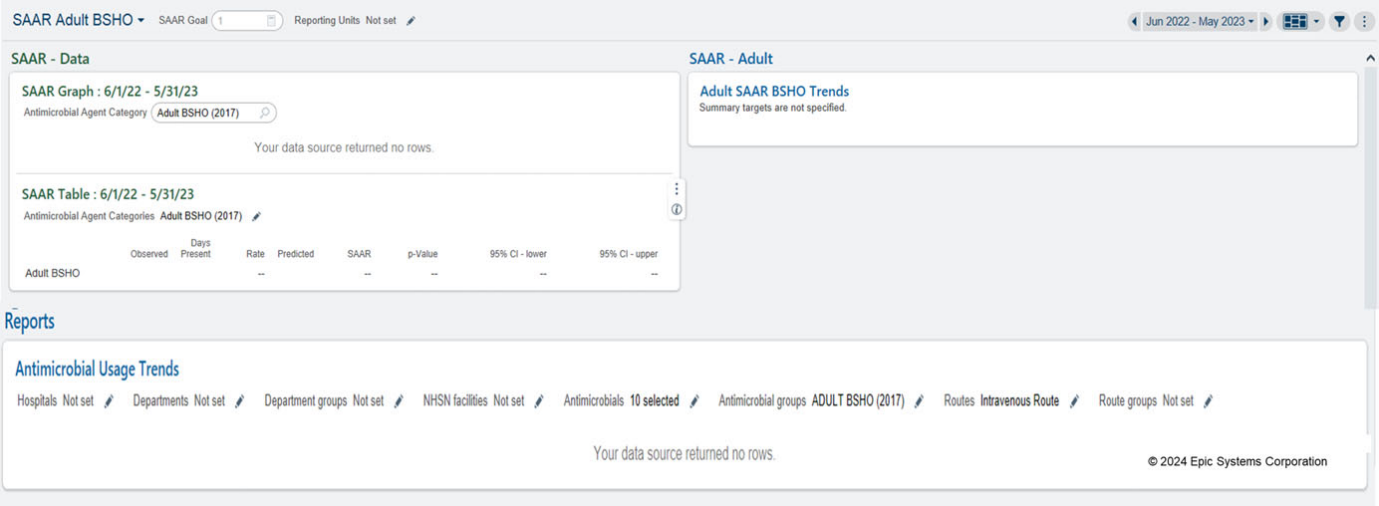
Default dashboard (BSHO pictured).

**Figure 2.**
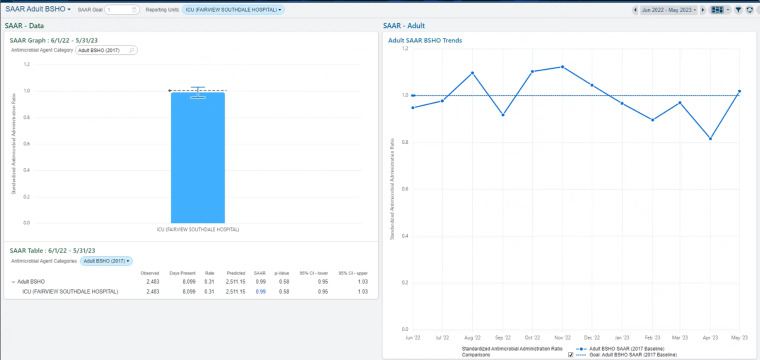
Dashboard with patient care unit selected, SAAR Section (BSHO pictured).

**Figure 3.**
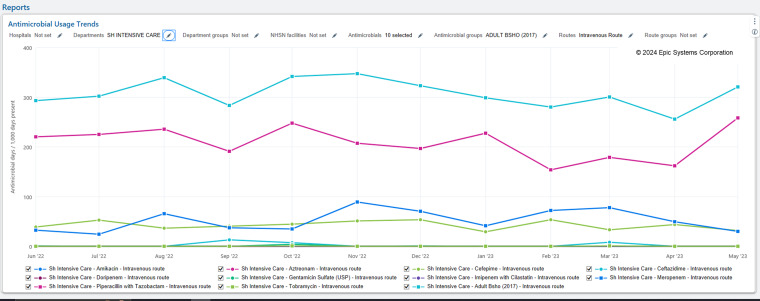
Dashboard with patient care units selected, AU Section (BSHO pictured).

The default configuration of the optimized dashboards is shown in Figure 1. To manipulate the dashboard, users select the pencil icon(s) and enter the variables they wish to modify. More details on dashboard functionality are described in detail in Appendix 1. The use of “2017” signifies the usage of the NHSN 2017 baseline SAAR predictive models, the most recent model for which the SAAR is calculated. Each dashboard visualizes the SAAR for the antimicrobial agent category as a trend over the last 12 months (Figure 2, “SAAR—Adult”) and the individual breakdown of the respective antimicrobials within the SAAR category according to the NHSN AUR protocol (Figure 3, “Antimicrobial Usage Trends”). Modifiable variables, including antimicrobials, hospitals, and patient care locations created by the EHR are preset to the corresponding antimicrobial agent categories for each dashboard. A user inputs the single variable of patient care location to retrieve data.

### SAAR section

The SAAR section is divided into three components, the graph, table, and trends. Figure 2 shows the SAAR section with the “SAAR Adult BSHO” selected at an intensive care unit within our health system. For example, this section shows in Figure 2 that this ICU had SAAR values >1 from October to December in 2022. The trends component shows how the SAAR has changed over time while the table component shows the values used to calculate the SAAR. The *P* value depicted in Figure 2 is for the 12 months to date, but selecting the SAAR will give a month-to-month table that shows the SAAR and *P* value for each month. Additionally, by selecting the SAAR value in the table component, the user can view a predicted number of days of therapy used to calculate the SAAR.

### AU section

The AU section (Figure 3) is preconfigured with the antimicrobials in the corresponding SAAR category dashboard. Additionally, a data point consisting of all the associated antimicrobials in the category representing a consolidation was created and is visible by default. Figure 3 shows the AU section of the dashboard, for the same location highlighted in Figure 2. The AU section displays the total days of therapy for the BSHO category using the BSHO grouper. The purpose of the grouper is to aid in visualizing which antimicrobials in the category are driving the SAAR. Piperacillin-tazobactam is the most utilized antimicrobial in the BSHO category in the selected department (intensive care unit).

## Discussion

### Significance of the optimized dashboard for healthcare professionals and patients

In this article, we describe a strategy to incorporate an EHR’s existing SAAR and AU modules into individualized dashboards by NSHN SAAR category for ASP utilization. Through the creation of this health-system SAAR and AU dashboard template, antimicrobial stewardship clinicians can assess the individual SAAR trends by category and the antibiotic DOT data that is contributing to an abnormal SAAR, in a singular view and in real time. This optimized dashboard offers two key benefits. First, it offers instant visualization of the DOT of interest, removing the potential for manual configuration. Second, it highlights the months where the SAAR remained unchanged but intra-category antimicrobial use might drastically change and allow for ASP action. For example, the BSHO category includes agents such as cefepime, piperacillin/tazobactam and meropenem, but categorizes them equally. If the SAAR and/or DOT for BSHO for a patient care unit is minimally or unchanged between months, but an increase in meropenem in place of less-broad anti-pseudomonal beta-lactams like piperacillin/tazobactam or cefepime occurs, this would be detected with the dashboard. For example, Figure 3 illustrates months when further investigation may be warranted. As piperacillin/tazobactam use declines, meropenem and cefepime almost always increases. From October 2022 to November 2022 and January 2023 to February 2023, the overall antimicrobial usage barely changes, but piperacillin/tazobactam usage decreases and meropenem usage almost doubles. This allows the ASP team to investigate the increase in meropenem for appropriateness. Like the SAAR section of the dashboard, the only variable the user must manipulate to display the data is the department (location of interest).

A standard operating procedure, Appendix 2, was created to integrate SAAR into our health-system’s stewardship program by requiring three elements. First, ASP site leads from each of the ten hospitals need to document their investigation and action for significantly abnormal SAAR’s for each antimicrobial category at the hospital level. These dashboards were made to aid in assisting with this expectation. Second, the ASP site leader coordinates with their infection prevention team to provide feedback on patient care locations during the assessment of NHSN location classifications. Finally, securing pharmacy IT analyst time and ongoing collaboration with ASP site leaders are essential to ensuring the dashboards are meeting the needs of the individual sites. While the customizations to the dashboard were built based on the needs of our pilot site, the next phase of these custom dashboards is the construction of custom dashboards for the remaining nine hospitals.

### Ongoing data validation

The custom dashboards were built to optimize efficiency and minimize maintenance for ASP pharmacists and IT resources as the AUR protocol and health-system evolve. As SAAR-eligible locations change and antimicrobial categories are updated by NHSN, the dashboards will need maintenance to reflect the changes. Additionally, patient care units are often changed to meet the needs of the health-system. If not accounted for when evaluating SAAR trends and operating our dashboards, inaccurate location mapping can lead to inappropriate and inaccurate data output and interpretation.

### Joint commission requirements

Our health-system plans to use the pharmacy IT analyst’s documented time spent creating and continued maintenance of these dashboards as supporting evidence to meet one of the updated elements of performance (EP 10.) under the MM.09.01.01 2023 TJC standards for antimicrobial stewardship.^9^ In addition, this work supports several other EPs under the MM.09.01.01 such as 14, 16 and 20. This work also supports elements of performance not under MM such as, performance improvement and infection control.

### Limitations of the dashboards

As the SAAR comes with multiple limitations, so do these dashboards.^4,12^ One of the greatest obstacles was deciding what route of administration to display on each dashboard for each antibiotic. For example, for the “g dashboard,” the default route “intravenous” was chosen. This is problematic, as the AU data display section of the dashboard then will not visualize enteral use of linezolid. If the route “any” or “enteral” are selected, oral vancomycin use is displayed on the AU data display, which should not be calculated in the resistant g infections SAAR category. While dashboards were built for adult and pediatric patients, neonatal SAAR data is not currently visualized due to the lack of validated benchmarking data from the CDC.^12,14^ In light of this, our team created location groupers for neonatal patient care units in anticipation neonatal SAAR data availability. Ideally, EHR providers would incorporate the entire NHSN framework (ie, adults, pediatrics, and neonates) into their antimicrobial usage modules. This is a significant build that will assist our ASP program in analyzing data more efficiently and lead to an increase in antimicrobial stewardship interventions. We recommend our customizations be a part of the EHR’s modules so the dashboard would be readily available for ASP utilization across the country.

This project was designed to be built and rolled-out in phases. Given the large size of our health-system, we opted to tackle our largest academic medical center data framework first as the complexity of the data was greatest. However, this project was initially scoped across our ten acute care hospitals. Phasing the build out allowed us to troubleshoot problems in the validation and implementation phases. Our pharmacy IT analyst was able to quickly make changes and re-validation could be completed in real-time. Now that the framework is built in our EHR, the SOP has been implemented, and pharmacy IT analyst time is secured, work is ongoing to build out the remainder of our acute care sites dashboards. We caution those seeking to mimic our dashboard customization strategy to ensure the role of pharmacy IT specialists, antimicrobial stewardship clinicians and infection prevention, as these team members were crucial to the development and roll-out of these dashboards. Notably, there is still maintenance associated with the dashboards, as when facility or unit characteristics are changed in NHSN, this is not automatically updated in Epic. Characteristic changes in NHSN must be manually made in Epic to ensure ongoing validity of the dashboards.

This optimized dashboard has the potential to elevate SAAR’s place in our health-system’s antimicrobial stewardship workflow and allows us to have defined standardized metrics across the system ASP. In addition, this dashboard visually is easy to follow and can be widely shared with hospital service line medical directors and senior and executive leaders within the organization. Other health-systems have shown SAAR can play a significant role at the regional and statewide level.^6^ Our work addresses Werth and colleague’s recommendation of improving analytical tools but is limited to peers with the same EHR.^7^ While we successfully optimized our SAAR dashboard, this should not be the expectation of our other ASP’s. We recommend EHR providers model their antimicrobial use modules to mirror that of the NHSN AUR protocol and the work of our system antimicrobial stewardship team. Additionally, Targeted Assessment for Antimicrobial Stewardship or TAS reports are incredibly valuable ways to translate SAAR data into actionable goals with metrics such as the ranked AU cumulative attributable difference or AU-CAD.^15^ These reports are available within the NHSN module. Unfortunately, there is no present method to visualize TAS framework data in the EHR dashboard we customized. While some features of dashboard do not require customization, our enhancements augment the already available data and visualization functionality. This study proves that customizable SAAR dashboards are reproducible for other ASP’s using Epic System’s Bugsy application and vital to improving the efficiency of daily ASP activities. Furthermore, EHR’s can incorporate a significant gap in the SAAR, patient level data.

## Conclusion

In conclusion, the optimization of the electronic health record’s default antimicrobial use dashboard into a dual-purpose dashboard to visualize SAAR and AU data simultaneously allows us to integrate the SAAR into our health-system’s antimicrobial stewardship program at both the site and system level. Other health-systems can look to our process as a resource for optimizing SAAR data visualization. Our work underscores the importance of collaboration between antimicrobial stewardship clinicians, infection preventionists and pharmacist information technology analysts in integrating the SAAR into antimicrobial stewardship programs. We recommend EHR providers use our custom dashboard as inspiration, as the data within our dashboards demonstrate what antimicrobial stewardship programs need to efficiently analyze NHSN AUR module data. Unless the EHR is enhanced, the dashboard cannot fully replace CDC SAAR reports. Many organizations lack the resources to build these features, but our work shows it’s possible within Epic’s base model. This is a call to mobilize partnership with EHR vendors to meet healthcare organizations to support this data model.

## Supporting information

10.1017/ash.2025.10050.sm001 Rondeau et al. supplementary material 1 Rondeau et al. supplementary material

10.1017/ash.2025.10050.sm002 Rondeau et al. supplementary material 2 Rondeau et al. supplementary material

## Supplementary material

For supplementary material/s referred to in this article, please visit https://doi.org/10.1017/ash.2025.10050
