# Supplementary material for: The fault in our SAAR: optimization and implementation of health-system dashboards for antimicrobial use and SAAR data
Source: Antimicrob Steward Healthc Epidemiol. 2025 Nov 5;5(1):e298. doi: 10.1017/ash.2025.10050 (PMC12616564; doi:10.1017/ash.2025.10050)
Supplement: Rondeau et al. supplementary material 1 — Rondeau et al. supplementary material [file S2732494X25100508sup001.pdf]

M Health Fairview
Antimicrobial Usage Report Guide

The purpose of this guide is to equip antimicrobial stewardship clinicians with the tools to integrate SAAR/AU data into their program management. The antimicrobial usage reports are now viewable in prepopulated, SAAR antimicrobial category dashboards, allowing ASP clinicians the ability to more rapidly investigate their antimicrobial usage data from a broad view and then zoom into individual units driving abnormal SAAR ratios. If a patient care unit is not eligible, or is incorrectly coded as one that cannot report a SAAR, these dashboards cannot be used to monitor their antimicrobial use as intended.

In order to maximize the usage of these dashboards, it is recommended you are familiar with the resources in the “NHSN SAAR Dashboard” folder. At minimum, users should be familiar with the following elements, outlined in the NHSN AUR Protocol, to utilize these dashboards effectively,

- What is a SAAR?
- How is a SAAR calculated?
- How to/not interpret a SAAR ratio (i.e. a SAAR <1 isn’t always a good thing)
- What locations are/aren’t able to generate SAAR’s?
- What are the SAAR antimicrobial categories?
- What antimicrobials compose each SAAR antimicrobial category?

These dashboards are expected to aid in identifying and driving the priorities of your antimicrobial stewardship program. They do not replace the performance of medication use evaluations, but can identify which evaluations to prioritize and provide points in your data to assess the degree of effectiveness of interventions generated from your evaluations. In pre-launch testing, high priority medication use evaluations were identified from these dashboards. Please consult the resources folder for guidance on implementing interventions based on NHSN AU data.

Accessing the Dashboards

1. Select the “Dashboards” tab and click where “Pharmacy Operational Dashboard” is to open the search bar.

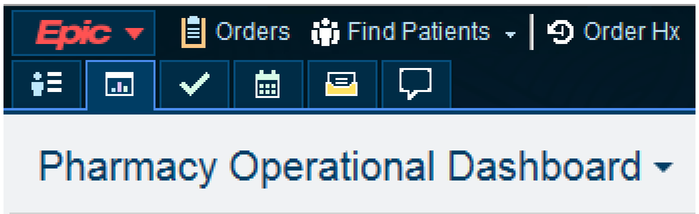

2. In the search bar, type “SAAR” and search

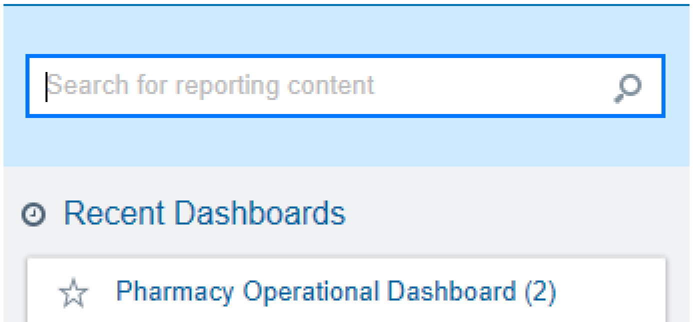

3. To the left and underneath the heading “Content Type”, select “Dashboards”. This will filter your search results to only show you dashboards. The components of the dashboards bear the same name and are pink images in your search results. The dashboards are blue images in your search results. Each SAAR dashboard is based on a SAAR antimicrobial category (i.e. BSHO, CDI, All antibacterial agents). You can favorite these dashboards, which will populate them in your “My Dashboards” tab.

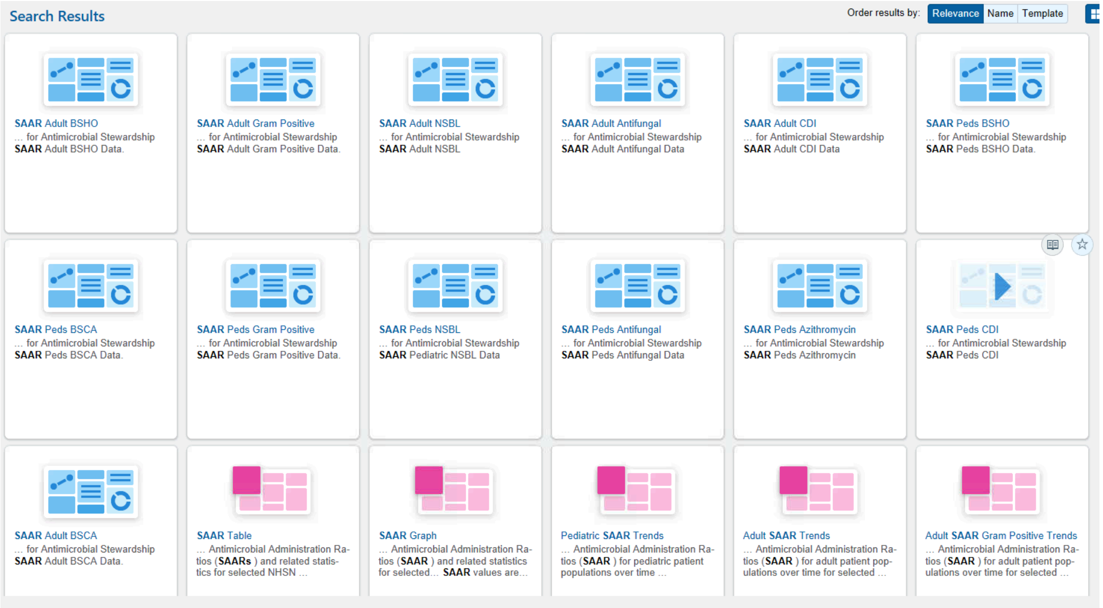

Manipulating a Dashboard

Each SAAR dashboard visualizes two items;

1. The SAAR for the antimicrobial agent category as a trend over the last 12 months
2. The antimicrobial usage (AU) of each agent within the SAAR category, represented by days of therapy (DOT)

The default configuration for each SAAR dashboard was designed to streamline answering the following question "If my SAAR is abnormal, what antibiotic(s) and which unit(s) are driving the abnormality." As you manipulate the dashboard, remember these tips if you get stuck or want to save your work.

A. Changes you make to the dashboards are NOT visible to other individuals.
B. If you close a dashboard, it will be just as you left it when you reopen it.
C. If you want to save how you've manipulated a dashboard, click on the below icon and then choose new view.

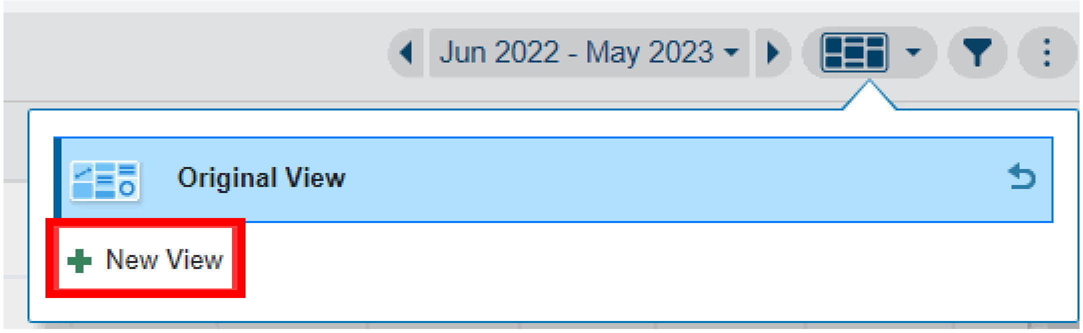

D. If you want to restore a dashboard back to it's default view, click on the below icon, then choose the "U-turn" arrow to the right of "Original View"

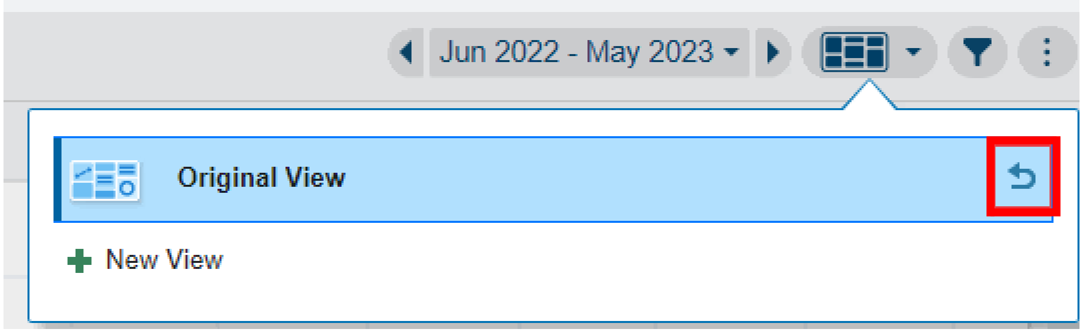

SAAR Section (Top Half)

The SAAR section is broken into three components, the graph, table and trends. All three of these components are controlled by the “Reporting Units” button depicted below. For this example, we’ll explore using the Adult BSHO dashboard for the ICU at Southdale. While this example is evaluating one unit, it can be used to visualize multiple units.

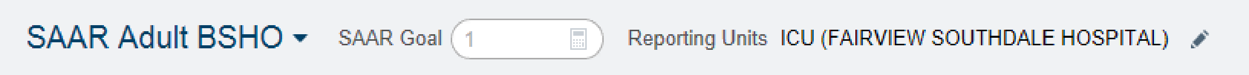

Below is the default configuration for the BSHO dashboard

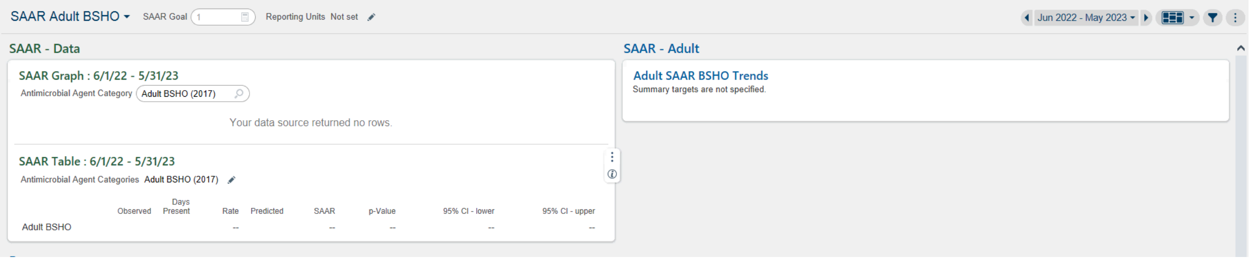

Now with Southdale’s ICU selected. Do note, there is not a standard nomenclature for units. If you are unable to find what your unit is called in the “Reporting Units”, consult the AUR location code document in the resource folder. If the unit you selected does not generate a SAAR, data will not populate on this screen and the SAAR table will report “No SAAR”. The most common reason for why “No SAAR” will appear is because the unit is not an eligible location type for SAAR calculations. Consult the AUR protocol for a list of eligible location types for SAAR calculations. Infection prevention manages changes to location types, therefore, discuss with your site’s infection preventionist to inquire/recommend a location type change.

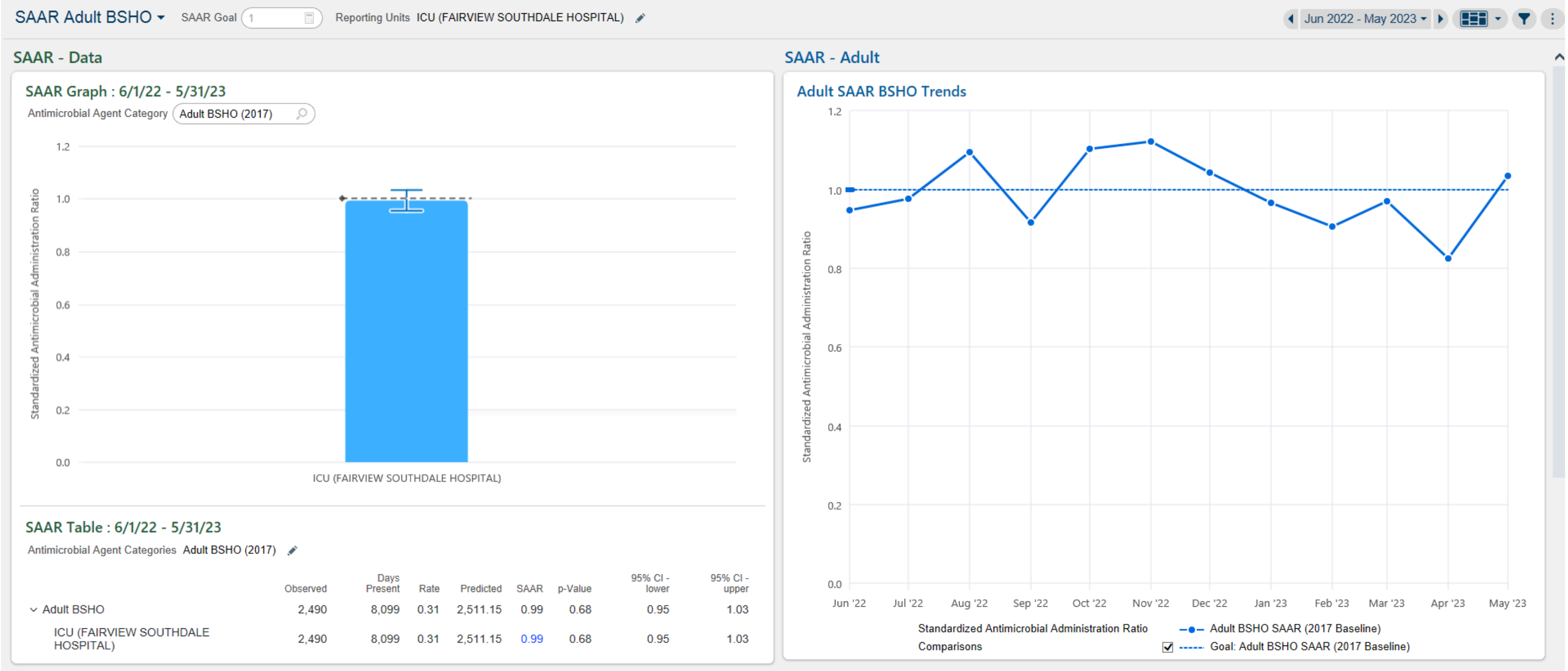

Last Reviewed June 2023

The SAAR graph shows you the SAAR for the named antimicrobial agent category, in this case BSHO, for the last 12 months for the selected unit(s)

The SAAR Table breaks down how the SAAR was calculated by showing the observed and predicted data. If multiple units are selected, **a cumulative SAAR will be displayed.** A quick way to see what the location type of your unit is can be seen by clicking the blue, linked number in the SAAR column of the table.

SAAR
0.99
0.99

ICU (FAIRVIEW SOUTHDALE HOSPITAL)

NHSN 2017 Baseline, Adult BSHO

Model Details

Patient Population	Adult
Antimicrobial Agent Category	Broad-spectrum antibacterial agents predominantly used for hospital-onset infections
List of Antimicrobials	Amikacin (IV only)<br>Aztreonam (IV only)<br>Cefepime<br>...(6 more)

SAAR Calculation

Observed Antimicrobial Days	Predicted Antimicrobial Days	SAAR
2,490	2,511.1494	0.99 (0.95 - 1.03) (Goal: 1.0000)

Facility Information

NHSN Facility Type	General Hospital, Including Acute, Trauma, and Teaching
Number of ICU Beds	41
Average Length of Hospital Stay	4.2 days
Educational Affiliation	Major Teaching

Location Information

NHSN Location Type	Medical-Surgical Critical Care

The SAAR trends is the most important piece of this section, as you will see in the second half of the dashboard the antimicrobials driving the trends mimic the SAAR trends.

We advise not changing the date/time from “month” to “quarter” unless the quarters you are viewing have completely passed. In testing, when viewing the data by quarters, the present quarter would report inaccurate SAARs.This is why the default configuration is “month” as it will show the most recent, complete month’s worth of data.

AU Section (Bottom Half)

The AU section is preconfigured with the antimicrobials that make up the corresponding SAAR in the selected dashboard. Additionally, a grouper of the associated antimicrobials in the category was built and defaults to being visualized in this section. If you notice the antimicrobial grouper and the SAAR trends look nearly identically, you're right! The purpose of the grouper is to aid in visualizing which of the antimicrobials in the category are driving the SAAR. Review the below tips before interpreting the AU section.

1. How do I change the time period pictured?
    a. Like the SAAR section, the time period visualized is defaulted to the last 12 months. If you want to change this, adjust the time period in the top right of the dashboard.
2. "I don't see anything"
    a. Remember the following recipe when data isn't populating "Location(s) + Antimicrobial(s) + Route(s)". If one of these isn't selected, your report won't generate anything.
3. What parameters should I use to manipulate the data?
    a. Hospitals
        i. Don't use this
    b. Departments
        i. This is where you go to choose which unit(s) you want to visualize
    c. Department groups
        i. If you want to visualize multiple units together, you can submit an IT ticket to have a grouper built for units of the same NHSN location type. Otherwise, don't use this.
    d. NHSN facilities
        i. Don't use this
    e. Antimicrobials
        i. This will be pre-populated based on the dashboard you selected. If you want to not have an antibiotic visualized, this is where you would go to deselect it.
    f. Antimicrobial groups
        i. This is where the grouper built for the antimicrobial category will be, if you don't want it to appear on the graph, go here to remove it.
    g. Routes
        i. This will be pre-selected based on the dashboard you choose. It is recommended to leave the default route configuration unless you are manipulating the Gram Positive SAAR dashboard.
    h. Route groups
        i. Don't use this

4. Why are there so many lines now it's so messy?
    a. For every variable you add to the AU section, i.e. another location, the number of data points can dramatically increase. If you want to visualize multiple units, we strongly recommend submitting an IT ticket requesting a location grouper be built for the unit of the same NHSN location type you wish to consolidate.

This is the default configuration of the AU section. Notice how the error "Your data source returned no rows" appeared. This is because a location or "department" hasn't been selected. Remember, you must have a complete "sentence" for the report to work. Again, that "sentence" is **"Location(s) + Antimicrobial(s) + Route(s)"**.

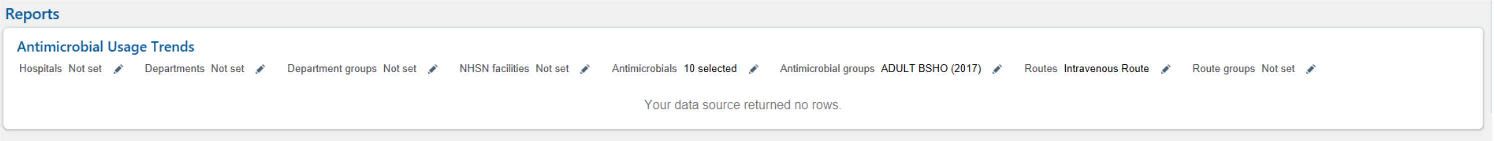

Remember that SAAR trend?

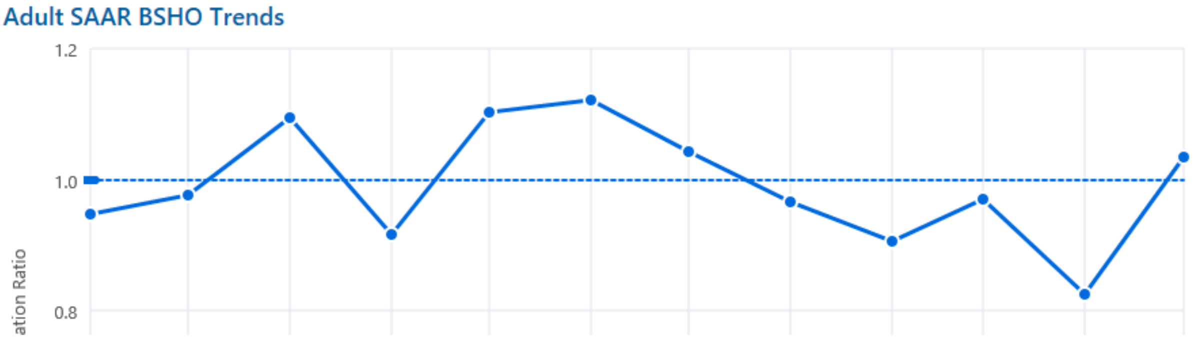

Now with the Southdale ICU selected, we can see what is driving the AU trend?

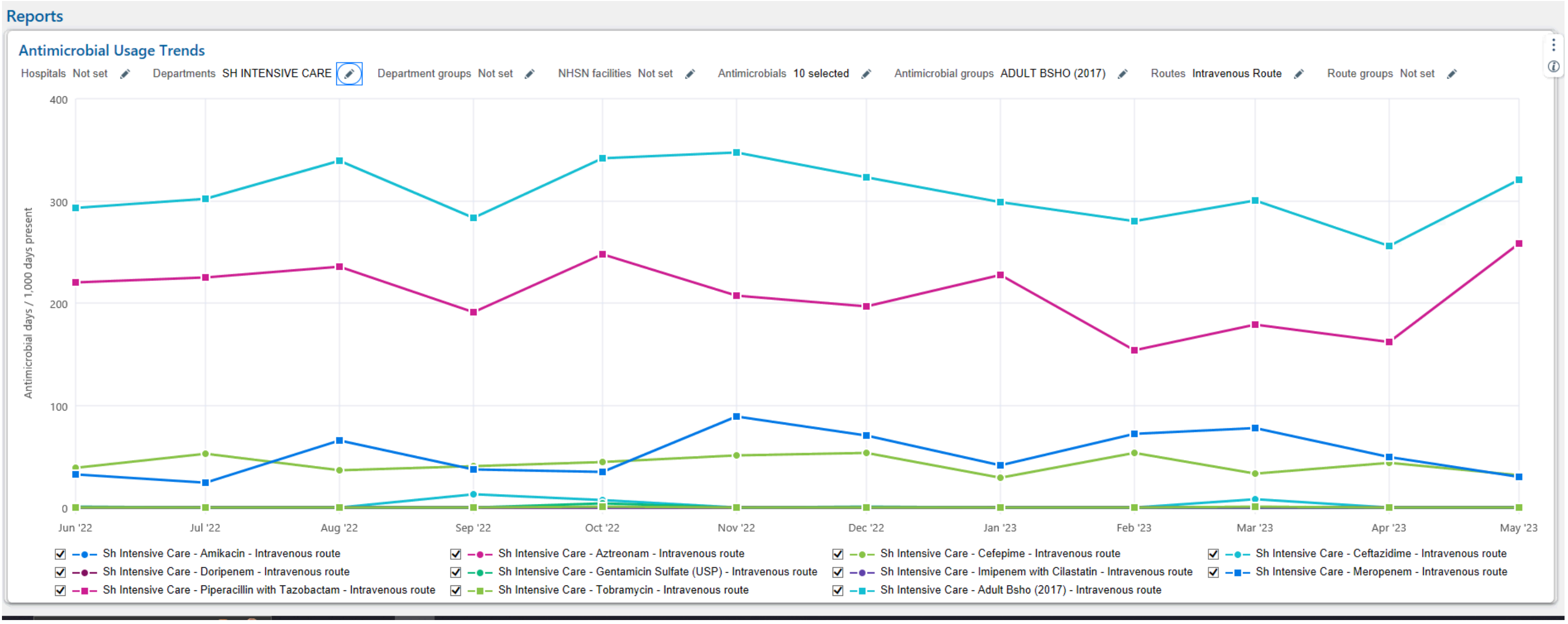

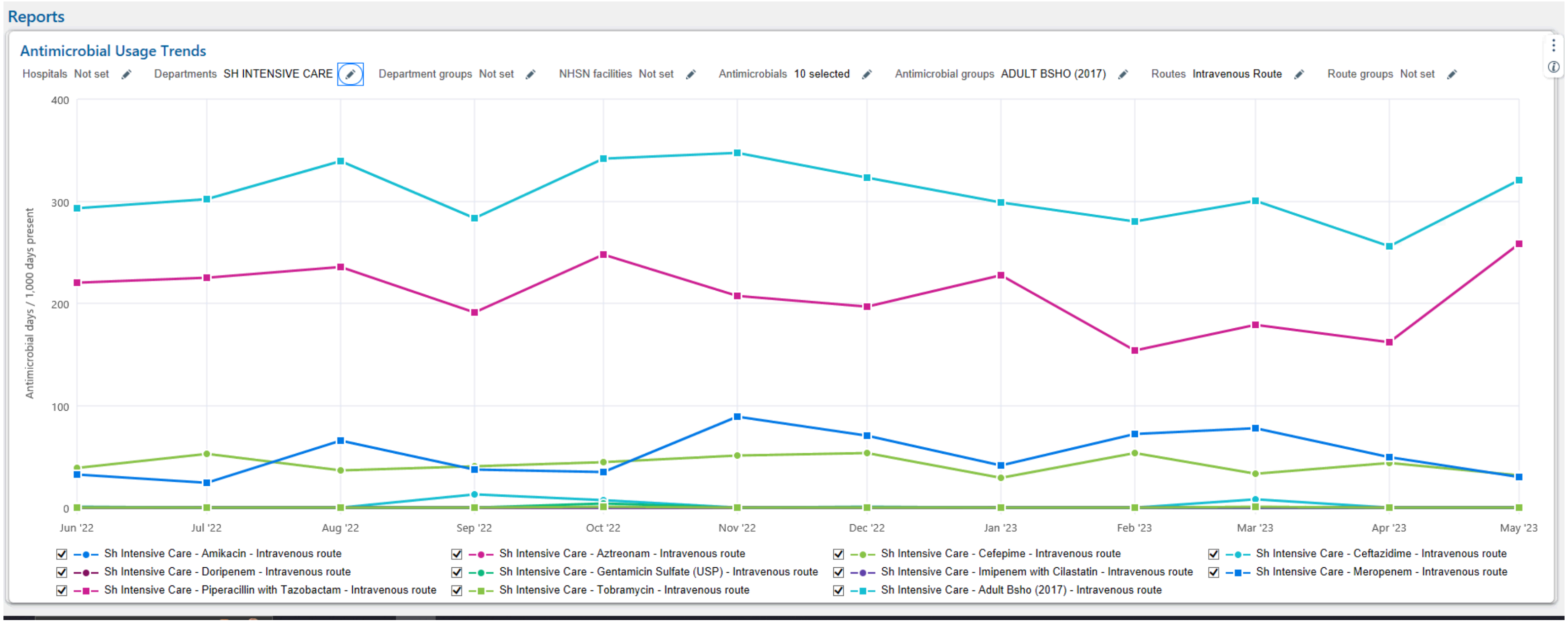

What you're seeing at the very top is the antimicrobial grouper, representing all the other antimicrobials of the BSHO antimicrobial category. A significant limitation of SAAR, specifically for this antimicrobial category, is the equal categorization of anti-pseudomonal beta-lactams and carbapenems. Using this dashboard, we can see piperacillin-tazobactam is the most commonly used BSHO antibiotic. In some months though, we see the overall antimicrobial use for the category increase while piperacillin-tazobactam decrease. October to November and January to February are both time points that prompt asking "Why did the overall antimicrobial use slightly change but my most commonly used BSHO antibiotic drastically fell?" Diving deeper, we see meropenem use increased in these time periods. Additionally, we can see that the largest spikes in AU are accompanied with an increase in the most commonly used BSHO antibiotic.

SAAR as an antimicrobial use benchmark has many limitations. Therefore these dashboards must be interpreted with caution and their use is not without significant limitations. Thus far, these are the major limitations we've identified.

1. The Gram-positive dashboard cannot be viewed with the route of "any" as it will inappropriately include oral vancomycin into the AU section of the dashboard. Until a solution is identified, we recommend visualizing this dashboard with the "intravenous route" selected or deselecting vancomycin and choosing "digestive route".
2. For units that are not eligible to generate a SAAR, the AU section of the dashboards can still be used to evaluate the antimicrobial use of the units. The NHSN location type may not be representative of the patient population cared for in the unit. Collaboration with Infection Prevention is key to ensuring SAAR's are appropriate representations of the units they are being calculated for.

Created by Hunter O. Rondeau PharmD PGY-2 ID Pharmacy Resident

Last Reviewed June 2023
