# Supplementary material for: The fault in our SAAR: optimization and implementation of health-system dashboards for antimicrobial use and SAAR data
Source: Antimicrob Steward Healthc Epidemiol. 2025 Nov 5;5(1):e298. doi: 10.1017/ash.2025.10050 (PMC12616564; doi:10.1017/ash.2025.10050)
Supplement: Rondeau et al. supplementary material 2 — Rondeau et al. supplementary material [file S2732494X25100508sup002.pdf]

Standard Operating Procedure for Quarterly Evaluation of NHSN SAAR Data

Purpose: The purpose of this standard operating procedure is to outline the steps to be taken by the Site and System members of the ASP Program to evaluate their National Healthcare Safety Network (NHSN) Standardized Antimicrobial Administration Ratio (SAAR) data quarterly.

Scope: This procedure applies to all healthcare facilities with antimicrobial stewardship programs that report to M Health Fairview's System Antimicrobial Stewardship Committee

Responsibilities:

1. The ASP Site Lead is responsible for leading the evaluation process and ensuring that NHSN's required data are collected and analyzed.
2. At least quarterly, the ASP site lead will document, in the form of an SBAR, statistically significant high SAAR ratios.
3. The site infection preventionist and site ASP lead are responsible for ensuring patient care units are accurately coded for the patient care population they service, taking into consideration the type of patient care units eligible for receiving SAAR reports.

Procedure:

Annually

1. The ASP site lead will provide feedback to site infection preventionist regarding the appropriateness of the NHSN location type of patient care units.
    a. If a unit has a statistically significant SAAR thought to be due to it's NHSN location type being an inaccurate representation of its patient population, documentation of a plan of action to more accurately represent it must occur
2. The System ASP lead and ASP site leads will request support from the Epic Willow team to ensure the SAAR dashboards are optimized for each site's use and updated with the latest guidance from NHSN

Quarterly

1. The ASP site lead will receive their SAAR reports from the system ASP lead.
2. The ASP site lead will compare the SAAR reports they receive from the System ASP lead to what is reported in the SAAR Dashboards.
3. For statistically significant SAAR ratios, the ASP site lead will identify areas for improvement based on their analysis of the data and document their assessment and plan of action.
    a. It is recommended to use the SAAR Dashboards available in Epic to aid in analyzing the SAAR data. Consult the tip sheet prior to use.
4. Implement the plan of action and monitor the effectiveness of the interventions.
5. Re-evaluate the NHSN SAAR data quarterly to assess the impact of the interventions and identify any new areas for improvement.
6. Document all steps of the evaluation process, including the findings, plan of action, interventions implemented, and outcomes, in the ASP Program's records.

Conclusion:

The ASP Program's quarterly evaluation of NHSN SAAR data is an essential component of an effective ASP program. This standard operating procedure provides a framework for the evaluation process, ensuring that all necessary data are collected and analyzed, and appropriate actions are taken to improve antimicrobial utilization and reduce the risk of antimicrobial resistance.

References

1. AU & SAAR Dashboard Tip Sheet
2. NHSN Annual AU Option Data Validation Protocol
